# Orchestrating faster access to products of non-profit R&D: a case study of a novel regimen for drug-resistant tuberculosis

**DOI:** 10.1136/bmjgh-2025-021596

**Published:** 2026-04-24

**Authors:** Suerie Moon, Marcela Cristina Fogaça Vieira, Kaitlin Large

**Affiliations:** 1 Global Health Centre, Graduate Institute of International and Development Studies, Geneva, GE, Switzerland

**Keywords:** Tuberculosis, Health Services Accessibility, Global Health, Treatment, Public Health

## Abstract

**Introduction:**

Non-profit product development partnerships (PDPs) have succeeded in bringing nearly 80 new drugs, vaccines and diagnostics for neglected diseases through regulatory approval, but arrangements to ensure they reach patients are unclear since the usual commercial incentives do not apply. We conducted a case study of how unusually fast access was achieved to a new treatment regimen for drug-resistant tuberculosis (DR-TB) developed by the TB Alliance (TBA). Over 100 countries procured the regimen in quantities to reach 67% of global demand by 2024, 5 years after first regulatory approval and 2 years after the WHO recommended it for routine use. What interventions contributed to this rapid rollout, and what role did the PDP play?

**Methods:**

We analysed the academic and grey literature, internal documents from TBA, and conducted interviews with 21 key informants from 16 organisations. We identified relevant interventions and actors, constructed a timeline and analysed the role TBA played.

**Results:**

We found TBA orchestrated a complex set of interventions implemented by dozens of actors over an 8-year time period across three categories: regulatory and normative guidance; market shaping for affordability and availability; and supporting country-level implementation through knowledge generation, knowledge sharing, stakeholder engagement and advocacy. Five attributes enabled TBA to do so: ability to generate and share knowledge about the product and regimen; non-profit status; ability to mobilise material resources for access interventions; pre-existing relationships and/or ability to develop new collaborative relationships; and intrinsic motivation to see the product reaching people with DR-TB.

**Conclusion:**

An orchestrator able to steer many actors towards the shared goal of reaching patients is critical in a complex ecosystem where no single organisation can realise access alone. Non-profit product developers can play this role well, including managing actual or perceived conflicts of interest, but require clearer mandates and financial support to do so.

**WHAT IS ALREADY KNOWN ON THIS TOPIC**

- The literature on non-profit pharmaceutical research and development (R&D) initiatives, such as product development partnerships for neglected diseases, has established that they are able to develop safe, effective products successfully. However, much less attention has been paid to whether and how patient access to such products can be delivered, in light of key differences with mainstream commercial incentives for marketing, distributing and selling new medicines.

**WHAT THIS STUDY ADDS**

- This study identifies the wide range of interventions required to realise patient access in a non-profit R&D model and the critical role and enabling attributes of an ‘orchestrator’ to steer a complex set of actors towards the shared goal of reaching patients.

**HOW THIS STUDY MIGHT AFFECT RESEARCH, PRACTICE OR POLICY**

- This study offers practitioners and policymakers a road map for how to address the many international, national and local-level hurdles to achieving patient access that may arise, even for new medicines that offer significant therapeutic and health system advantages and that were developed to be affordable, available and appropriate for use in limited-resource settings.

## Introduction

 At the turn of the millennium, concerns were rising about the failure of the mainstream market-driven pharmaceutical research and development (R&D) system to develop medicines for diseases that predominantly affected the world’s poorest populations, referred to as the neglected tropical diseases (NTDs).^1^ This prompted the creation of about two dozen public-private product development partnerships (PDPs) to boost innovative efforts into these diseases.^2^ While PDPs vary in how they operate, they are generally non-profit organisations with independent legal identities, funded mainly by public and philanthropic sources, designed to advance R&D for health technologies targeting unmet health needs and prioritising health outcomes rather than market returns.^3^ By 2023, PDPs had successfully obtained regulatory approval for 79 new drugs, vaccines, vector control products and diagnostics, including a drug and diagnostics for tuberculosis (TB).^4^ However, since PDPs largely focused on product development, arrangements were unclear for how these new medicines could reach patients, as the usual commercial incentives for doing so did not apply. Although there is a growing body of literature on PDPs, it has largely focused on R&D.^5^ Recent scholarship has discussed access for PDP-developed products,^6 7^ but systematic analyses of PDPs’ access strategies remain scarce.

For several health conditions that have been a high global health priority (ie, malaria, TB, HIV), ad hoc arrangements have emerged among the ecosystem of global actors (eg, United States Agency for International Development, Global Fund, Unitaid, Global Financing Facility and international national government organisations) to facilitate access. For many NTDs, such as sleeping sickness, dengue fever or schistosomiasis, there is no dedicated set of actors or playbook to facilitate access, exacerbating the substantial risk that products reach patients after long delays, with lives lost, suffering prolonged and money wasted.

It is, therefore, instructive to analyse a successful example of how access was achieved for a PDP-developed product. The recently developed TB drug, pretomanid, was developed by the non-profit PDP TB Alliance (TBA) as part of a new regimen to treat drug-resistant (DR) TB, comprised of bedaquiline, pretomanid and linezolid, with or without moxifloxacin (BPaL/M). (‘BPaL/M’ indicates the regimen may be used with or without moxifloxacin (M); for brevity, we use BPaL/M throughout this article except in cases where the addition or subtraction of moxifloxacin is of substantive importance, such as when calculating regimen prices.) TBA took a new approach by developing pretomanid as part of a regimen with bedaquiline and linezolid—two relatively new drugs without prior resistance to TB—rather than as a standalone drug. This regimen approach offered the important advantage that evidence on how pretomanid could be used with other drugs was available immediately on regulatory approval, averting the need for additional trials to answer this key question—one factor that contributed to faster uptake compared with bedaquiline and delamanid.^8^

**Figure 1:**
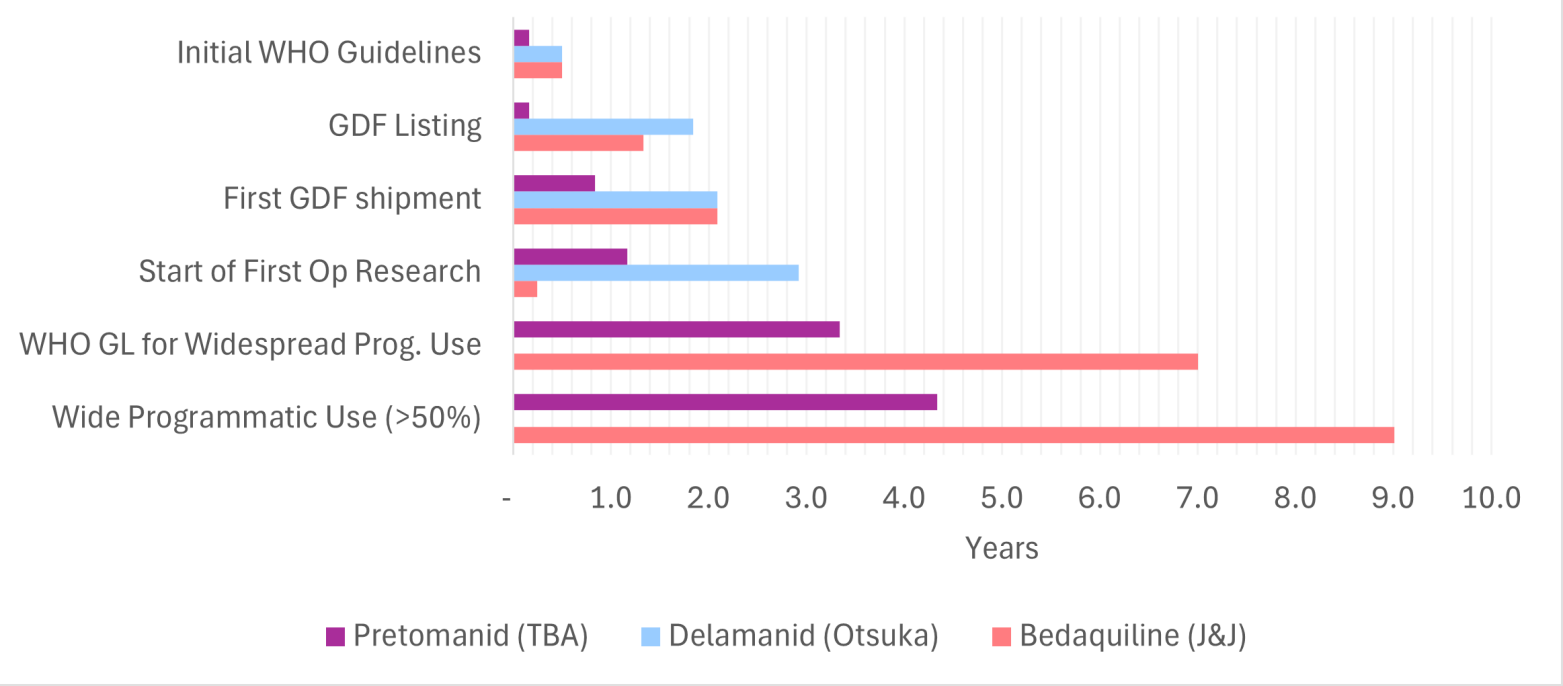
Years after first SRA approval (data and figure source: TBA). GDF, Global Drug Facility; GL, guideline; SRA, stringent regulatory authority; TBA,Tuberculosis Alliance.

In 2024, over 100 countries had procured BPaL/M in quantities to reach about 109 000 patients or 67% of global demand (~164 000 people were treated for DR-TB that year), 5 years after obtaining first regulatory approval^9 10^ (see figure 1) and less than 2 years after the WHO recommended it for programmatic (routine) use. What interventions contributed to such rapid uptake, and what role did the PDP play?

This study describes the wide range of interventions adopted to accelerate access to BPaL/M, analyses the role of TBA and identifies potential lessons for how to ensure rapid access to other products of non-profit R&D. It contributes to the literature on PDPs by focusing on access strategies and on the literature on BPaL/M, complementing evidence on safety, efficacy, acceptability and cost-effectiveness by analysing how rapid access was achieved globally.

## Methods

We reviewed the academic and grey literature and internal documents provided by TBA. We conducted semistructured interviews with 21 participants from 16 organisations primarily between July and September 2024, using snowball and purposive sampling. We purposefully selected interviewees to represent different parts of the ecosystem of stakeholders involved in the uptake of BPaL/M, including funders (n=4), guideline developers (n=1), technical assistance providers (n=4), researchers (n=1), national TB programmes (NTP) (n=1), community members and civil society (n=3), TBA and other PDPs (n=6) and manufacturers (n=1). Interviews were conducted in English, lasted between 45 and 90 min and took place online via video conference platforms. One interview was conducted via email. Interview guides were tailored to each type of interviewee, and questions asked pertained to the strategies adopted and specific roles that each stakeholder played in facilitating access to BPaL/M. With the informed consent of each interviewee, interviews were recorded and transcribed with Otter.ai software and transcripts lightly edited for clarity or to preserve anonymity. Interview transcripts were only seen by the three researchers and not shared with TBA. TBA employees were interviewed, shared internal documents, and provided valuable comments on earlier drafts of a research report^11^ from which this article is derived, which helped to ensure its factual accuracy and completeness; the analysis and final conclusions remain the product and responsibility of the authors. Dissemination plans for this research include presentations to community groups, including patients and the public.

We analysed interview data according to three thematic areas of activity identified inductively: (1) regulatory and normative guidance, (2) market shaping for affordability and availability and (3) country-level implementation support, with a cross-thematic focus on the role of TBA. All interviewees had an opportunity to review a draft of the research report and their quotes and provide clarifying remarks.^11^

This study has three main limitations. First, due to time and resource constraints, interviews were carried out within a short time period, and it was not feasible to interview all relevant stakeholders. We sought to mitigate data gaps by including a wide range of organisational roles, but interviews with a broader set of national stakeholders, including NTPs, guideline developers and regulators, would provide a more complete dataset. Second, the initial list of interviewees was provided by TBA and may not represent the full breadth of perspectives. Relatedly, our position as researchers conducting a study with financing from TBA may have elicited biased responses from interviewees or biased our own interpretations. We sought to mitigate bias by encouraging frank responses to questions by ensuring anonymity of the interviewees and by identifying and interviewing additional key informants beyond the initial list. Of the 21 interviewees from 16 organisations conducted for this study, 15 interviewees from 12 organisations came from the initial TBA list. We believe the study provides a reasonably complete picture of the actions TBA took to support access to BPaL/M and the role it played vis-à-vis other actors, a key objective. However, it does not provide a full account of all interventions undertaken by all contributing actors, which would require a far more extensive study. Third, we did not examine how access was handled for other non-BPaL/M regimens for DR-TB (eg, delamanid-based), nor did we analyse the uptake of new treatments for drug-sensitive TB or other products developed by PDPs, which were beyond scope. Future research that addresses these limitations would enrich our understanding of various approaches to improving access to newer TB treatment in particular and to the products of non-profit R&D more broadly.

## Results

We found TBA orchestrated a complex set of interventions implemented by dozens of actors over an 8-year time period across three categories.

### Regulatory and normative guidance

#### Regulatory approvals

TBA licensed pretomanid from biotech company PathoGenesis in 2002 and subsequently conducted several studies involving the drug, including the pivotal trial that enabled it to file for first regulatory approval with the US Food and Drug Administration (FDA). TBA chose to submit to a stringent regulatory authority (SRA) on the rationale that it would give many stakeholders confidence in the regimen’s safety and efficacy. (We use the term SRA in this paper as it was the terminology used when pretomanid was submitted for approval, while recognising that WHO has transitioned its terminology to ‘WHO-listed authorities (WLA)’.^12^) In 2007, pretomanid had received orphan drug and fast-track designations from the US FDA, which granted it expedited review, a registration fee waiver, and ‘a streamlined clinical development program,’ which ‘may involve smaller, shorter or fewer clinical trials’.^13^ TBA also received a priority review voucher (PRV) as part of the approval process by the US FDA in acknowledgement of the significant unmet medical need in the field of DR-TB. Regular communication with the US FDA provided input on the kinds of data and trial design required to meet its standards. The US FDA approved pretomanid in August 2019 as part of a combination regimen with bedaquiline and linezolid for treating adults (above 14 years old) with certain forms of highly drug-resistant pulmonary TB.^14^

SRA approval would also quickly unlock eligibility for countries to use international donor funds to purchase the new regimen. Many countries receiving funds from the Global Fund to Fight AIDS, TB and Malaria (GFATM) procure their TB medicines and diagnostic tools through the Stop TB Partnership’s Global Drug Facility (GDF). The GFATM and GDF require that any medicines or diagnostics it finances must either be prequalified by WHO, approved by an SRA or the GFATM’s Expert Review Panel. TBA licensed pretomanid to five manufacturers. Their licensing agreements required manufacturers to have approval from an SRA or WHO Prequalification (PQ) for export. In November 2020, Mylan (now Viatris), the first TBA-licensed manufacturer for pretomanid, received abridged WHO PQ based on the US FDA approval.^15^

In addition, medicines typically also need to be registered with national regulatory authorities (NRAs) for distribution in any given country. In some countries and situations, it is possible to obtain a registration waiver, especially for medicines procured through donor-linked procurement mechanisms such as GDF, but not in all. TBA agreed on a priority list of countries where licensees would fast-track filings, particularly where such waivers were difficult or where national budgets (not donors) purchase drugs. TBA also met regularly with licensees to track progress on registration, supported them in responding to regulatory queries and participated in meetings with regulators if needed. By late 2024, pretomanid had been approved by 33 regulatory agencies for use in 62 countries.^10^

#### Normative guidance

TBA also engaged informally with WHO on how the new regimen could be incorporated into its TB treatment guidelines. Unlike the US FDA, there were no formal processes for product developers to seek, or for WHO to provide, information on the kinds of evidence required. WHO recommendations can profoundly influence NTPs and are often a requirement for international donor funding.

Shortly after US FDA approval, WHO issued a rapid communication in December 2019 flagging to countries that it expected to recommend pretomanid-containing regimens for XDR-TB treatment, in order to accelerate transitions at the country level.^16^ However, the Guidelines Development Group (GDG) deemed evidence from the first trial (Nix-TB) insufficient for recommending routine use worldwide.^17 18^ Nix-TB was a Phase 3 single-arm study conducted by TBA at three sites in South Africa with 109 people.^19 20^ The GDG raised concerns about the relatively small number of people enrolled, the limited evidence provided by this trial, and the fact it was not a randomised controlled trial, which together limited the generalisability of the study findings to all populations and all regions.^17 18^ Thus, in May 2020, WHO recommended BPaL for XDR-TB under operational research conditions^21^ until additional evidence could be generated.^22^

Nix-TB had demonstrated 90% efficacy of BPaL but also a high incidence of adverse events attributed to the linezolid dose. Therefore, even before the FDA decision or WHO’s assessment, TBA started the ZeNix trial in 2017 to test lower linezolid dosing. It found the high efficacy of BPaL could be maintained while reducing linezolid dosing and its associated adverse events.^23 24^

At the same time, Médecins Sans Frontières (MSF) conducted another trial studying BPaL (TB-PRACTECAL), for which TBA provided input on trial design and pretomanid supply. The trial showed that BPaL/M performed better than the standard of care while being much shorter with a lower pill burden and fewer adverse events.^25 26^

**Figure 2:**
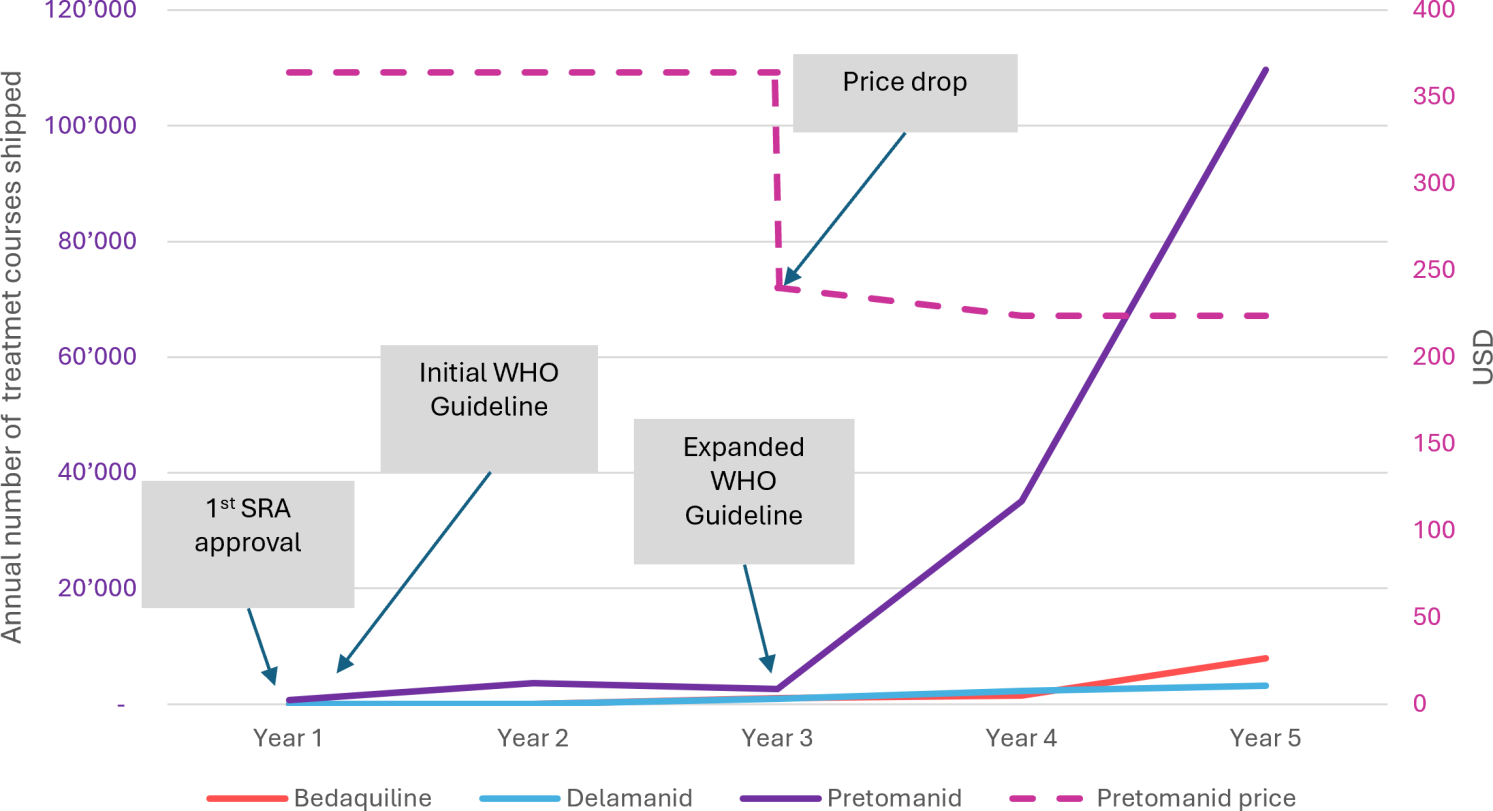
Number of pretomanid treatment courses shipped since first SRA approval (data and figure source: TBA). SRA, stringent regulatory authority; TBA, Tuberculosis Alliance.

With additional evidence from ZeNix and TB-PRACTECAL, the WHO issued a rapid communication in May 2022 greenlighting programmatic use of BPaL/M for DR-TB and in December 2022 incorporated this recommendation into its comprehensive revised guidelines.^22 27^ This recommendation unleashed an upsurge of demand (see figure 2). Furthermore, TBA’s application for adding pretomanid to WHO’s Essential Medicines List (EML) succeeded in 2023.^28^ The WHO EML informs many national EMLs, which can unlock domestic funding and accelerate uptake.

In parallel, TBA worked with NTPs and where needed, technical partners, to help align national guidelines with WHO guidelines and add pretomanid to national EMLs. By late 2024, 24 of the 30 highest MDR-TB burden countries had implemented or decided to implement BPaL/M.^10^

Some interviewees suggested that the broader 2022 WHO recommendation could have been obtained faster with a different clinical trial or regulatory strategy that would have averted the need for operational research. As noted, TBA’s rationale for filing first with the USFDA was to permit rapid use of donor funds and because of the approval’s weight with other NRAs. Because pre-XDR-TB was rare in the US, the US FDA allowed for ‘smaller, shorter or fewer clinical trials’ to approve pretomanid.^13^ This strategy may have accelerated the timeline to first SRA approval, but did not generate evidence WHO considered sufficient for global guidelines applicable to TB high-burden countries.

It seems that the timeline from a conditional (2019) to full (2022) WHO recommendation could have been shortened with earlier evidence generation through a multicountry RCT with a larger total sample size. Doing so would imply a different regulatory strategy and could have required a longer initial trial and/or much more funding. An alternative would have been to seek first approval from a TB high-burden country’s national regulator^29^ which could have required a different trial design and generated enough evidence to obtain WHO recommendation for programmatic use from the start. However, none of these were an SRA or WLA,^12^ and many national regulators have limited experience reviewing new chemical entities. Seeking first regulatory approval from a TB high-burden country would therefore have restricted eligibility to procure medicines with international funding, which many high-burden countries rely on for their TB programmes. In other words, there were important trade-offs regarding speed, degree of evidence and access to international funding between choosing to submit to an SRA vs a high TB-burden NRA for the first approval.

Interviewees also flagged the need for clearer understanding between the product developer and WHO on its evidentiary requirements for recommending routine use of a new medicine. In December 2024, WHO published guidance on the kinds of evidence needed for its TB treatment guidelines.^30^

### Market shaping for affordability and availability

TBA orchestrated various interventions towards making BPaL/M affordable and available, recognising that the regimen’s clinical benefits alone would not ensure country adoption.

#### Non-exclusive licensing to quality generic manufacturers

As with most PDPs, TBA did not have in-house manufacturing facilities. To ensure production and spur competition, TBA granted non-exclusive licences for the low and middle-income country market to three generic producers with track records of quality production, starting with Viatris (then named Mylan) in 2019^31^ then Macleods several months later^32^ and Lupin in 2021^33^. Licences were also agreed with two other generic manufacturers in China (Hongqi in 2020^34^) and Pakistan (Remington in 2023^35^), a strategy to facilitate product uptake and security of supply primarily in those countries. Despite multiple non-exclusive licences, Viatris was the only supplier with SRA/WHO PQ through October 2024 and therefore held a de facto monopoly for nearly 4 years in the donor-funded market. Macleods obtained WHO PQ in October 2024 and Lupin in September 2025.

#### Price negotiation and volume guarantee

**Figure 3:**
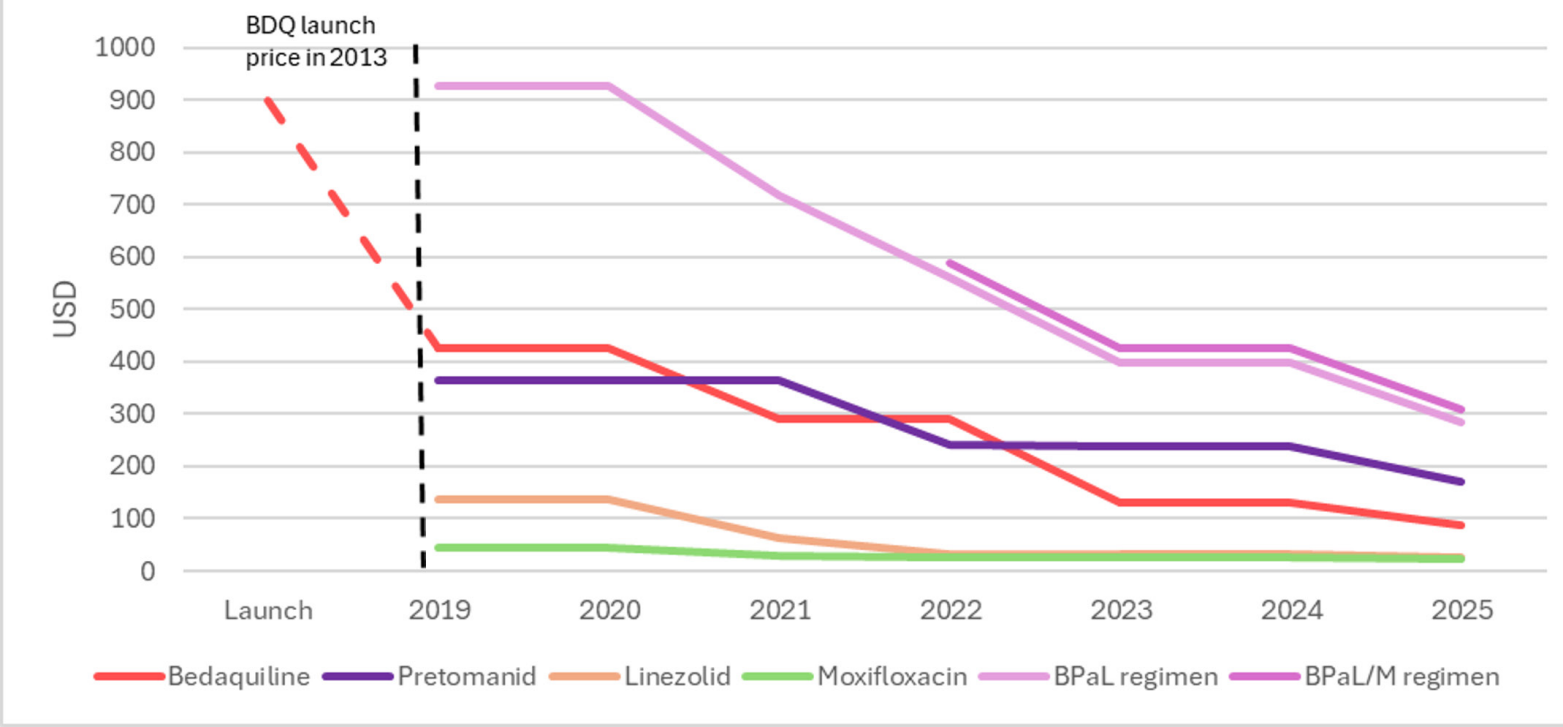
Evolution of BPaL/M price (data and figure source: TBA). BDQ, bedaquiline; BPaL/M, bedaquiline, pretomanid and linezolid, with or without moxifloxacin; TBA, Tuberculosis Alliance.

TBA negotiated a launch price of USD 364 for pretomanid with Viatris, the lowest launch price of a novel TB drug.^36 37^ BPaL’s initial price (~US$1000)^38^ was significantly lower than some prior DR-TB regimens (US$2000–US$8000),^39^ but still exceeded the target price civil society groups advocated (US$500)^40^ based on production cost estimates.^41^ In 2022, anticipating increased demand from the broader WHO recommendation, TBA helped broker a volume guarantee between the global health financing vehicle MedAccess and Viatris, who agreed to cap the price of pretomanid at US$240.^42^ The volume guarantee insulated Viatris from the risk of revenue losses in case volumes did not materialise, while enabling it to lower prices to facilitate increased demand. By 2023, price reductions in all three components of BPaL dropped the regimen to below US$400, then to US$310 in 2025^43^,^45^ (see figure 3).

#### Economic evidence for decision-makers

TBA invested in economic analyses to demonstrate that adopting BPaL/M was financially feasible and advantageous for countries. To facilitate national-level decision-making, TBA published acceptability/feasibility, cost-savings, budget impact, and cost-effectiveness studies for BPaL/M, and engaged in demand forecasting with NTPs and manufacturers.^46^,^50^ For example, a TBA-commissioned study on the budgetary impact of adopting the new regimen in Indonesia, Kyrgyzstan and Nigeria found cost savings of 57%-78% compared with the conventional regimens and that a gradual adoption of BPaL/M would result in 5-year savings of 15%-32% of the national budgets to treat XDR-TB.^51^ Another TBA-commissioned study in South Africa, Georgia and the Philippines found that NTPs could save 64%–70% by using BPaL/M over the standard of care and avert an additional 46%–56% of disability-adjusted life-years (DALYs).^52^

TBA also developed an online tool for countries to estimate their own cost savings (‘Savings from Leveraging & Adopting Shorter & Highly Effective TB Treatments’), helping to remove budgetary uncertainty as a barrier to uptake. A study on the tool in Pakistan, the Philippines, South Africa and Ukraine found cost savings, increases in lives saved, treatment success and DALYs averted in each country.^53^

Furthermore, TBA projected demand for DR-TB treatment in 13 high-burden countries to estimate the use of BPaL/M globally, useful for planning production and estimating price evolution.^54^

Together, these studies and tools generated evidence that supported the rapid adoption of BPaL/M.

#### GDF listing and GFATM advice

TBA ensured rapid inclusion of pretomanid into existing global procurement channels. Notably, GDF added pretomanid to its catalogue 2 months after US FDA approval, the fastest inclusion of any TB drug post-SRA approval.^38^ In July 2022, shortly after WHO’s rapid communication recommending programmatic use, the GFATM started advising countries to consider transitioning to BPaL/M.^55^

#### Concerns flagged

However, economies of scale were difficult to achieve for the relatively low-volume DR-TB market, a challenge for ensuring supply security and low prices. This issue is also relevant for other small markets, such as many NTDs and paediatric formulations. When pretomanid was first approved for pre-XDR TB in 2019, only about 12 000 people worldwide were on treatment with 9–18 month regimens.^56^ Once pretomanid was recommended for MDR-TB, the market expanded to around 178 000.^56^ Compared with HIV, with nearly 20 million persons on lifelong treatment, volumes for XDR and MDR-TB were much less attractive to producers (especially alongside pricing pressure), making it challenging to secure manufacturers. Relying on one supplier may offer sufficient profit to keep them in the market for the long term, whereas multiple suppliers provide increased competition and security of supply—but may not remain in the market if returns are low.

Views differed on how to balance trade-offs between the benefits of multiple suppliers (ie, competition, security of supply) against the risks (ie, fragmentation, market exit) in a low-volume market. Some interviewees also called for TBA’s licences to be published to improve transparency and facilitate price negotiations^57 58^ and for better coordination between TBA, GDF, WHO and manufacturers to avert shortages, as uncertainties of supply were raised by interviewees as reasons for countries not to adopt BPaL/M-based regimens sooner.

Despite these challenges, BPaL has undergone consistent and substantial price cuts over time, contributing to rapid uptake.

#### Supporting country-level implementation through knowledge generation, knowledge sharing, stakeholder engagement and advocacy

The third major area of TBA’s orchestration was country implementation. Working with a diverse group of countries, TBA co-created and shared knowledge on how BPaL/M could be implemented on the ground, thereby building capacities to switch to the new regimens.

#### Operational research and pilot projects

Prior to the first regulatory approval, TBA commissioned an acceptability and feasibility study in three representative countries with varying burdens of MDR-TB, concluding that there was a high likelihood (88%) that the treatment would be adopted once available, especially given its patient friendliness and potential to reduce burden on the healthcare system.^59^

Following the 2019 WHO conditional recommendation, in October 2020 TBA launched the project ‘Leveraging Innovation for Faster Treatment of Tuberculosis’ (LIFT-TB) to conduct operational research in Indonesia, Kyrgyzstan, Myanmar, the Philippines, Ukraine, Uzbekistan and Vietnam, co-funded by TBA and the Korea International Cooperation Agency. TBA also contracted international technical assistance providers such as the Koninklijke Nederlandse Centrale Vereniging tot bestrijding der Tuberculose (KNCV) and local partners to execute operational research in project countries, including engaging TB-affected communities.^60^ Studies also took place in other countries, both directly with TBA and independently. For example, TBA supported a pilot of BPaL in the high-burden country of Pakistan, with 116 patients treated in two provinces under normal programmatic conditions. Data on outcomes, clinical and programmatic experiences was collected, supporting scale-up.^61^ Furthermore, in December 2020, South Africa launched its BPaL/M Clinical Access Program, funded by the USAID and run in partnership with the Wits Health Consortium.^62^

Proactive involvement of country-level and global stakeholders in producing evidence generated early buy-in from several high DR-TB burden countries, who became early adopters of the regimen.

#### Guidance and tools for implementation

To help ensure learning from early experience, TBA contributed to developing a BPaL implementation guide in collaboration with partners in South Africa^63^ and funded the creation of country-specific implementation plans in several countries. By co-creating these plans with health ministries and TB experts in-country, TBA contributed to local ownership and readiness. TBA also supported knowledge-sharing through WHO’s BPaL Accelerator Platform, a global forum where countries and experts regularly convened virtually to share experiences, protocols and troubleshooting tips for BPaL implementation.^64^ Separately, it established the PeerLINC Knowledge Hub with partners in the Philippines, a peer-to-peer channel for technical assistance directly from and to those implementing programmes.^65^

#### Stakeholder engagement and advocacy

Furthermore, TBA engaged TB-affected communities to develop independent feedback loops within their NTP. Hearing patient experiences and community perspectives helped address concerns and maintain pressure for rapid uptake. In 2023, when WHO and partners released a high-level ‘Call to Action’ urging countries to adopt new DR-TB treatments, TBA also launched the Fast Track the Cure project, which mobilised communities at the grassroots level to amplify this call.^66^

The fruits of these country-engagement strategies are reflected in 24 of the 30 high DR-TB-burden countries implementing or committing to implement BPaL/M as of late 2024.^10^

## Conclusions

**Figure 4:**
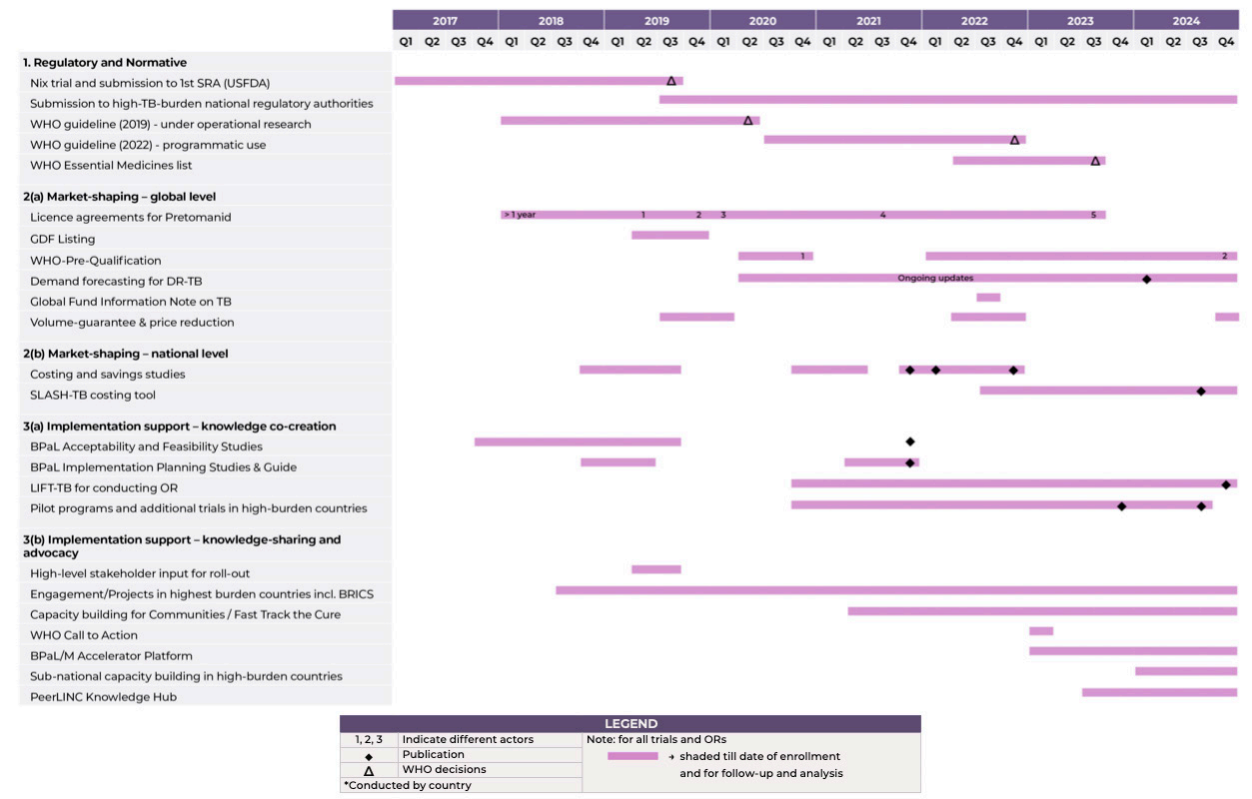
Timeline of access interventions orchestrated by TBA. GDF, Global Drug Facility; LIFT-TB, Leveraging Innovation for Faster Treatment of Tuberculosis; OR, operational research; SLASH-TB, Savings from Leveraging & Adopting Shorter & Highly Effective TB Treatments; SRA, stringent regulatory authority; TBA, Tuberculosis Alliance; USFDA, US Food and Drug Administration.

TBA orchestrated a wide range of interventions with partners over 8 years, working in parallel at global, national and subnational levels (see figure 4). While TBA did not deliver services directly, it was able to orchestrate, that is, ‘enlist public or private intermediary actors on a voluntary basis, by providing them ideational and material support’ to pursue their goals.^67^ It is a particularly relevant strategy for global actors who often do not have hierarchical authority to command or decide what others do, but enlist the cooperation of other actors to achieve their goals.

**Figure 5:**
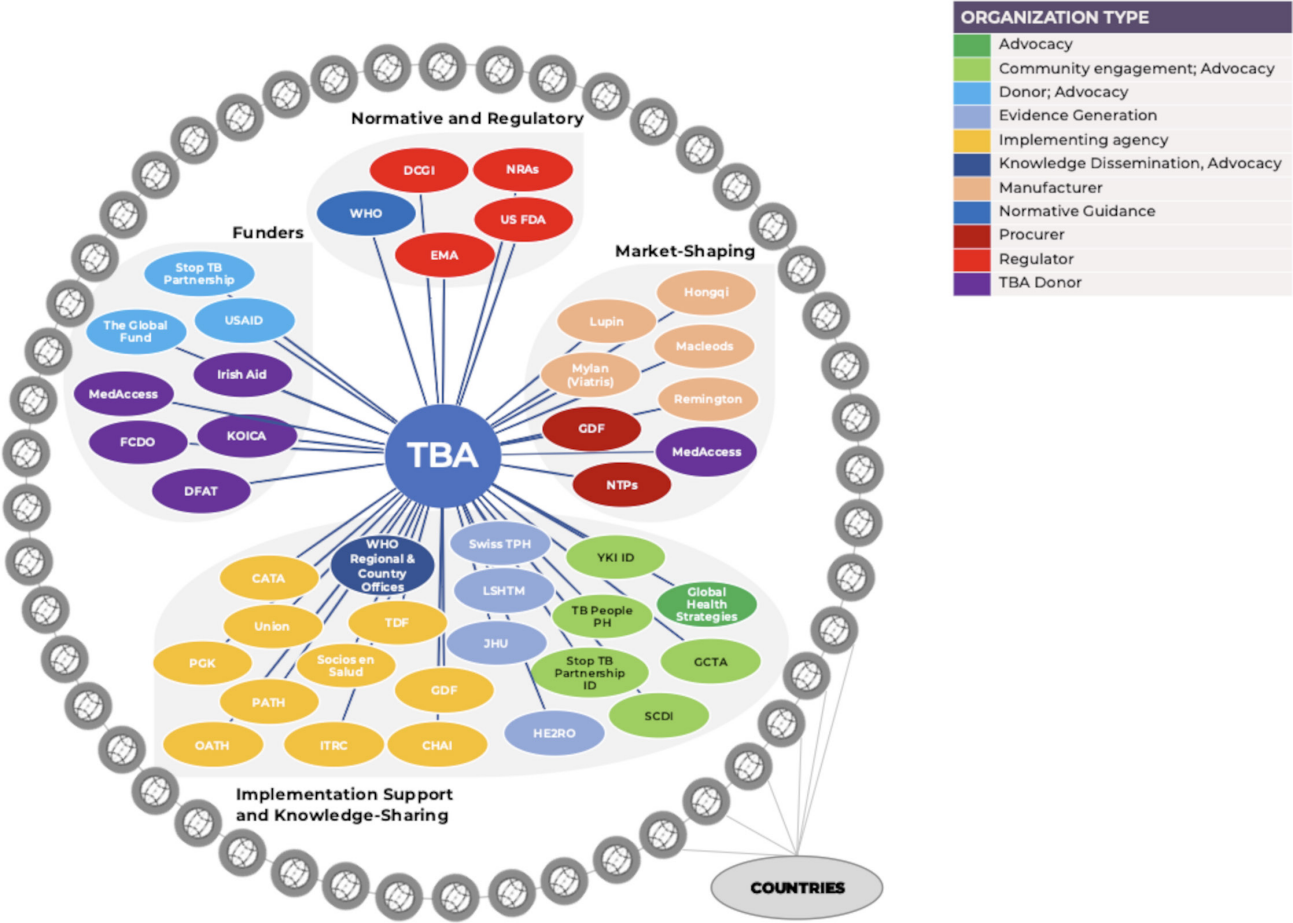
TBA orchestration of access interventions with partners. CATA, Chinese Anti-Tuberculosis Agency, China; CHAI, Clinton Health Access Initiative, USA; DCGI, Drugs Controller General of India, India; DFAT, Department of Foreign Affairs and Trade, Australia; EMA, European Medicines Agency; GCTA, Global Coalition of TB Advocates; GDF, Global Drug Facility; HE2RO, Health Economics and Epidemiology Research Office, South Africa; ITRC, International Tuberculosis Research Centre, Republic of Korea; JHPIEGO, Johns Hopkins Program for International Education in Gynecology and Obstetrics, USA; KOICA, Korea International Cooperation Agency, Republic of Korea; LSHTM, London School of Hygiene and Tropical Medicine, United Kingdom; NRAs, national regulatory authorities; NTPs, national TB programmes; OATH, Organization for Appropriate Technologies in Health, Ukraine; PATH, Program for Appropriate Technology in Health, USA; PGK, Pyi Gyi Khin, Myanmar; TDF, Tropical Disease Foundation, Philippines; SCDI, The Centre for Supporting Community Development Initiatives, Vietnam; TBA, TB Alliance, USA; USAID, United States Agency for International Development; US FDA, US Food and Drug Administration; YKI ID, Yayasan KNCV, Indonesia.

We identified five ‘ideational and material’ attributes that enabled TBA to orchestrate a wide range of actors effectively (see figure 5).

1. Ability to generate and share knowledge about the product and regimen: As the developer, TBA’s in-depth knowledge of BPaL positioned it well to provide authoritative information quickly to regulators, WHO, NTP managers, clinicians and community members. Co-creation of field evidence then generated comfort and buy-in among countries.
2. Non-profit status strengthened TBA’s legitimacy to orchestrate other actors: It is difficult to imagine a for-profit firm engaging in the range of activities orchestrated by TBA, either because it would face insufficient incentive to do so or because stakeholders would not accept it. In contrast, TBA’s non-profit status allowed it to engage as a public health actor with other not-for-profit actors such as community groups, NTPs, regulators and WHO.
3. Ability to mobilise material resources for access interventions, either by funding activities directly or by pro-actively raising new funds: While TBA’s donors had expected the organisation to focus primarily on R&D, it had received flexible core funding that allowed it to finance at least some smaller-scale access activities early on. For larger-scale access activities, TBA raised new funds, for example, from the Republic of Korea for LIFT-TB and from Australia for PeerLINC. For some interventions, TBA cooperated with other actors, such as GFATM, WHO or MSF, who could contribute their own financial resources to the shared objective of expanding access to BPaL/M.
4. Pre-existing relationships and/or ability to develop new collaborative relationships with a wide range of actors: Many of these relationships originated in the product development process, such as with the regulators, WHO and manufacturers. Others became a focus as attention shifted to access, such as with the NTPs, GFATM, new donors, in-country technical assistance contractors, community groups and advocacy organisations.
5. Intrinsic motivation to see the product reaching people with DR-TB, a key indicator of how well TBA was achieving its non-profit organisational mission: TBA’s mission motivated an energetic, creative and diverse set of interventions that successfully promoted rapid uptake of BPaL/M.

Many PDPs have most if not all of these attributes—or could develop them—making them a natural fit to orchestrate. The main counterargument is that they may have a conflict of interest (CoI) in doing so. PDPs do not have the same types of financial CoIs as for-profit firms, but they do have an organisational interest in seeing their products widely used.

Views differed on whether such organisational interests comprised a conflict, and if so, how to define and limit the product developer’s role in key policy decisions such as regulatory, WHO guidelines and national adoption. While none of the interviewees expressed concerns that TBA in particular had unduly influenced policy decisions, it remains a general issue that merits attention. Interviewees also suggested that if the product developer is involved in evidence generation or technical assistance, transparency regarding its role and partnering with other organisations is important to mitigate potential CoIs.

Conversely, the non-profit product developer’s organisational interest in seeing its product used widely can be a valuable motivator when this interest is aligned with public health objectives. Enduring motivation is necessary to do the hard work of mobilising financial, human and organisational resources to continue putting in place intervention after intervention until medicines have reached those who need them. We conclude that finding acceptable ways to manage potential CoIs is critical, as in some cases there may not be other actors beyond the PDP with the motivation or other attributes necessary to orchestrate access.

The BPaL/M case offers broad lessons for ensuring that products emerging from non-profit R&D quickly reach those in need. First, generating evidence simultaneously for regulatory approval and normative guidance by designing pivotal trials to provide evidence sufficient for both regulators and national or WHO guidelines could reduce the usual delay between these stages. Formalising the existing informal exchanges between WHO and product developers would facilitate mutual understanding on the kinds of evidence needed. In the medium term, wider adoption among WHO Listed Regulatory Authorities and SRAs of processes to assess a product for use by populations other than their own may be needed; for example, the European Medicines Agency collaborates with national regulators of disease-endemic countries to issue a scientific opinion on a medicine that will be used outside the European Union. In the longer-term, investments in strengthening national regulatory capacity in high-TB-burden countries are merited. Furthermore, improvements in the WHO PQ process and timelines could also reduce the relative need for SRA approval for international procurement.

Second, even in cases of therapeutic game-changers, realising access requires a strategy to address economic considerations of decision-makers at both global and national levels and to make cost savings to health systems visible.

Third, TBA began building understanding of how to implement BPaL/M in NTPs by conducting acceptability, feasibility and implementation studies even prior to regulatory approval. Participating in clinical trials, operational research and pilot projects built the foundation in many countries for rapid uptake. For other products of non-profit R&D, early and sustained country engagement seems critical to build knowledge on implementation.

Finally, an orchestrator able to steer many actors towards the shared goal of reaching patients is critical in a complex ecosystem where no single organisation can realise access alone. If non-profit R&D actors are to orchestrate access effectively, they must be supported by clear decisions mandating access as part of their organisational missions, with political and financial support from boards and funders.

With financing of PDPs shifting away from flexible core funding to become increasingly earmarked for specific (mainly R&D) projects, it may become more difficult for PDPs to implement access interventions early—unless funders provide resources to do so. PDPs have historically been financed primarily through official development assistance (ODA) and philanthropic foundations, but ODA from traditional donor governments is now under severe strain. Funders who have invested in successful non-profit R&D have a stake in getting the job done and should consider providing sufficient resources for access interventions. In the longer term, other sources of funding are likely to be required. One option is to generate funding from alternative sources, such as the US PRV, a programme that grants the developer of NTD (and other) products a voucher for priority regulatory review that can be sold to a commercial firm. PDPs have obtained PRVs, including TBA for pretomanid, and they can provide flexible resources for R&D and access activities, though the programme has significant loopholes.^68^ Another option is for endemic countries to finance a greater proportion of access interventions, not only within their own borders but also for the global-level orchestration that facilitates national-level access.

The rapid uptake of BPaL/M makes it a highly instructive case, but the generalisability of these findings is limited by several factors. First, BPaL/M was a major improvement over the standard of care, and it may be more difficult to achieve such rapid uptake for more incremental improvements. Health technology assessments will become even more important to make the case for new products. Second, there were many international actors with the mission to support TB treatment, but this is not the case for most NTDs, for which new products are most likely to emerge from non-profit R&D. This raises the question of how existing actors can best organise themselves to achieve access and who can play the orchestrator role. Finally, the literature on non-profit R&D has focused on innovation, but further research on access to its end products is needed.

Despite these limits, the BPaL/M case is a remarkable success story, as one interviewee simply stated:

The introduction of BPaL/M–we have not seen something like that before, the way countries have rapidly implemented this recommendation!

The case points the way towards ensuring that the growing arsenal of products emerging from non-profit R&D can quickly reach those whose lives are on the line and cannot afford to wait.

## Data Availability

Data on timelines, prices, volumes, interventions and actors involved are included as an Excel file in the public open access repository Zenodo here: https://zenodo.org/records/18069938. Interview data are not made available, in order to protect the anonymity of interviewees.
